# Utilization and efficacy of DotPhrases in the electronic medical record for improving physician documentation

**DOI:** 10.1080/08998280.2024.2352993

**Published:** 2024-05-20

**Authors:** Palak A. Fichadia, Mini Virmani, Priyanshi Shah, Ramsha Mahmood, Rhea Kanwar, Amishi Singla, Rohit Jain

**Affiliations:** a Department of Medicine, Smt. N.H.L Municipal Medical College, Gujarat, India; b Department of Quality Improvement, Penn Medicine Princeton Medical Center, Plainsboro Township, New Jersey, USA; c Department of Oncology, Mayo Clinic, Rochester, Minnesota, USA; d Avalon University School of Medicine, Willemstad, Curaçao; e Pennsylvania State University College of Medicine, Hershey, Pennsylvania, USA; f Dallastown Area High School, York, Pennsylvania, USA; g Department of Internal Medicine, Penn State Milton S. Hershey Medical Center, Hershey, Pennsylvania, USA

**Keywords:** DotPhrases, electronic health records, health information management, patient safety, smart phrases

## Abstract

Clinical documentation of patient visits has changed over the last 20 years, with the increasing use of electronic records causing a seismic shift in how notes are taken. Electronic note-taking aims at reducing the time taken to document a visit, and the introduction of dot phrases, or DotPhrases, in electronic medical records is a step toward reducing the time required to update patients’ charts, which might allow doctors to spend more time with their patients. DotPhrases, abbreviated phrases used in the electronic medical record, help in the simplification of note-taking and the standardization of notes. They also allow for a more comprehensive note from physicians and ensure that no information is undocumented. On the contrary, however, excessive usage of DotPhrases can lead to an excessively long and cumbersome note. This can overwhelm physicians and lead to them missing crucial information that is buried somewhere in the notes. Although there is ample research studying the benefits of DotPhrases, adequate research must also be carried out to understand their shortcomings and disadvantages. This article aims to shed some light on use of DotPhrases and to outline their advantages and disadvantages affecting patient management and care.

Accurate and comprehensive clinical documentation of patient visits forms the backbone of the clinical, insurance, and medicolegal worlds, providing a thorough summary of the diagnosis as well as the treatment.^1^ Clinical notes are especially important in the emergency department, where many patients first encounter a hospital. To improve the quality of health care administration, diagnostic accuracy, and patient safety, there have been a large number of advances in information technology in the health care sector.^2^ These advancements have reduced redundancies and streamlined billing administration.^3^ As a result, the US health care system has made the switch to the electronic medical record (EMR) one of its chief objectives.^4^

Despite the increasing importance of the EMR, there is no ideal standardization of all documentation, making note-taking and interpretation much harder. This lack of standardization increases the time required to produce high-quality notes. Consequently, there have been many strategies and innovations to combat this issue. For instance, a substantial number of health care practices utilize medical scribes, dictation software, or personalized EMR documentation shortcuts.^5^ Most EMR vendors allow these shortcuts, known as DotPhrases, called SmartPhrases in Epic, Patient Objects in CPRS, and AutoText in Cerner. DotPhrases is a feature within the clinical note that allows users to add commonly used information into the note or discharge summary. DotPhrases are used to enter text quickly by increasing consistency and reducing the number of abbreviations, misspellings, and grammatical errors. Starting with a period and expanding into a larger body of text upon typing them into notes, DotPhrases are extremely flexible.^6^ Therefore, DotPhrases are one of the most used tools for charting because they create structured documentation that is of high quality while simultaneously reducing the burden of documentation and maintaining the flow of language in text. Any individual with medical training, spanning from attendings to medical students to allied health professionals, such as nurse practitioners or physical therapists, can create DotPhrases.^7^ Additionally, DotPhrases can be shared, leading to simplification of work because users do not have to generate their own phrases from scratch. A generalized set of DotPhrases could even be established for a clinical practice. Examples are provided in *Table 1*.

**Table 1.** Examples of DotPhrases used in various software

DotPhrase	Macro text
a1chigher	Your average blood sugar (A1C) is higher than it was last time. It rose from ____% to ____ %.
Lipidcvdrisk	Your total cholesterol is ____. It should be less than 200. Your good (HDL) cholesterol is ____. It should be more than 40. Your bad (LDL) cholesterol is ____. It should be less than 100. Your triglycerides (fat dissolved in your blood) is ____. It should be less than 150. Your LDL (bad) cholesterol is/is not where it should be. Continue your current treatment. Exercising, eating low-fat, low animal–fat foods, and losing weight will help improve your cholesterol numbers. You have a ____ % chance of having a heart attack or stroke in the next 10 years. The American Heart Association guidelines recommend that you take a cholesterol-lowering medicine (statin) to lower this risk. I want you to start taking ______.
Backpain	Take the anti-inflammatory medicine (naproxen, ibuprofen) as prescribed. Take the steroid pills (prednisone) each morning with food. Read the handouts I gave you and start the back stretches. Once your back pain starts to get better, start the back exercises.
Cin	Will call if normal and call for follow-up appointment if abnormal.
Odrisk	The patient was screened for opioid use disorder or risk for opioid overdose and provided appropriate counseling.

Though associated with a multitude of benefits for users, DotPhrases also have drawbacks. Excessive use of DotPhrases leads to cumbersome and potentially even unmanageably long notes.^8^ In addition, because of the flexibility of individuals to create personalized DotPhrases, phrases that are minimally used or made by users who no longer work in the institution may be forgotten. This article examines the use of DotPhrases in documentation of patient visits and their impact on efficiency and note quality.

## ADVANTAGES OF DOTPHRASES

DotPhrases have become much more common in EMRs in recent years, benefiting doctors in several ways. The following sections present a few advantages of DotPhrases. Advantages as well as disadvantages are summarized in *Table 2*.

**Table 2.** Advantages and disadvantages observed with DotPhrases

Category	Examples
Advantages	Personalization of notes using DotPhrases Faster completion of notes Homogeneity and standardization of notes Effective communication between the care team and improved care effectiveness Ease in data gathering Improved quality and patient safety Improved patient satisfaction Better billing documentation
Disadvantages	Overdocumentation and time consumption Difficulty in health information management Increased clinical decision support fatigue Negative impact on advance care planning Increased claims and billing

### Personalization and faster completion

Through using DotPhrases, clinicians can customize health record templates to fit their preferences and workflow, which has been linked to higher EMR satisfaction.^9^ Furthermore, the literature has demonstrated that DotPhrases decrease documentation time. A cross-sectional study was performed in a 150-bed children’s hospital and involved 39 pediatric residents; they developed standardized, efficient note templates. The templates had a few autopopulated sections (like vital signs), and they were made to encourage creative, autonomous input through free-text fields (like assessment/plan and overnight events) and text prompts (like a list of organ systems under the physical examination section). According to the resident poll, 78% of participants stated that the new note form was simpler to use and allowed for quicker note completion.^10^ Similar studies have shown that providing residents with standard note templates and teaching them best practices for documenting—like keeping lists to a minimum and encouraging original ideas—could save documentation time while simultaneously enhancing the quality of the notes.^11^ Because the note-writing strategy outlined here allows for early note-taking to prevent losing important details, avoids after-hours documentation work, and allows for interaction with the patient during the visit, it accomplishes the goal of reducing burnout.^12^ In a study using DotPhrases and voice recording, 100 notes were recorded at an outpatient clinic. This resulted in doctors finishing notes on time and taking less than an hour to write 10 to 20 notes, which increased their job satisfaction. Clinicians liked the completed notes’ professional and well-formatted appearance.^13^

### Homogeneity and standardization

Additionally, there is a considerable degree of homogeneity in the notes made using these DotPhrases, which boosts their trustworthiness and preserves a consistent quality, even in the face of diverse writing styles and user patterns for EMRs. Additionally, it appeared that this held true for both sexes and for subspecialties.^14^ By employing standardized note templates, the physician trainee gains a better understanding of the fundamentals of documenting the patient experience. Although these electronic phrases have been passed down by residents, gathering and integrating them into workflows may help bring the EMR to a new level.^15^ An instance of how clinicians might employ DotPhrases is by using the EMR to boost referrals and awareness for preexposure prophylaxis (PrEP). To do this, new DotPhrases regarding PrEP offerings can be created using a single question (e.g., “Are you interested in hearing more about a pill that can prevent HIV?”) or existing DotPhrases can be expanded upon by providers (e.g., for general health care maintenance, sexual health risk screening, or sexually transmitted infection treatment and follow-up).^16^ Bennett and Steen demonstrated how disease-specific EMR templates with clickable inputs improve clinical record quality and billing and reimbursement by increasing the number of completed charts within 30 days.^17^

### Communication and care effectiveness

DotPhrases have also proved to be helpful as part of the multimodal approaches (e.g., targeted education and awareness, real-time reminder sites, monthly email feedback, and incremental EMR improvements to retrieve data automatically) that can be implemented to produce a significant improvement in the medical records system.^18^ “Problem-oriented assessment and plan” progress notes were suggested in pediatric hospital research involving 22 residents, promoting thorough patient coverage of patient complaints and action plans. The problem list functioned as a gauge for the efficacy of email communications, helping providers become more conscious of their performance and modify their work methods.^18^

### Data gathering

One practical method of gathering data is through DotPhrase data collection. Paper data forms are no longer necessary because these data forms save all study data in a secure EMR environment. In a study performed over 1 year, it was noted that data entry errors were less likely when DotPhrase forms were used as compared to paper forms.^4^

### Quality and patient safety

On a systemic level, using standardized hand-offs with the help of DotPhrases has been shown to improve 30-day hospital all-cause readmissions. This was seen at a local dialysis center with 6 months of follow-up in patients with end-stage renal disease, where DotPhrases were used in 95.4% of the discharge summaries, showing its contribution to patient safety.^19^

## DISADVANTAGES OF DOTPHRASES

On the contrary, DotPhrases have unique drawbacks that can limit their utility in the EMR. In the following sections we outline some of these disadvantages. By investigating these points, we can work toward improving the existing technological infrastructure in health care.

### Increased paperwork and time consumption

The amount of paperwork that is now routinely completed at every medical visit has increased dramatically, taking up between 25% and 50% of a doctor’s time. Patient care is hampered by the time-consuming nature of data collection. The amount of time a doctor has to spend with patients and their families, as well as for teaching and clinical research, is diminished by the time spent on reviewing and finishing paperwork.^20^ DotPhrases can help expedite clinical documentation, but they do not solve the problem of overdocumentation and can still result in long, ambiguous, and inconsistent notes.

### Health information management

Retrieving pertinent patient information from a large volume of potentially redundant and irrelevant material is time-consuming.^21^ A lack of uniformity in clinical documentation results from patients seeing many doctors, each of whom may use a different DotPhrase. The fact that DotPhrases may remain in the EMR long after a provider leaves the health system and no other provider utilizes the stored DotPhrase is another inefficiency that has been seen.

### Clinical decision support fatigue

Doctors’ fatigue with clinical decision support (CDS) is another problem. A systemic inability to react to warnings and reminders is a hallmark of CDS tiredness, according to a University of Utah study. This study showed that the EMR system is not intended to assist physicians’ workflow and decision making. CDS fatigue includes both alert tiredness and exhaustion concerning clinical reminders. CDS has a variety of causes, such as an inconsistent workflow in the clinicians’ schedules, and DotPhrases could further exacerbate physicians’ fatigue.^8^ The main reason is the excessively long length of notes, causing physicians to spend more time at their computers, which inadvertently leads to increased fatigue.^8^

### Potential impact on advance care planning

When DotPhrases were employed, several gaps were found in a retrospective review study examining the use of EMR in advanced care planning (ACP) documentation. First, ACP rulings do not have a single, recognized venue. Because hospitalists or physicians based in hospitals can access EpicCare to view documentation created in the outpatient setting, having uniform locations facilitates the expeditious retrieval of such documentation in an inpatient scenario. There is not much time for doctors in the emergency department or intensive care unit to make decisions. An “accident of timing” could lead to these doctors not following the patient’s wishes if the ACP choice was hard to find at the point of service. The second issue is the progress note regarding ACP talks may not be correctly signed. Even if a doctor takes the time to search rulings in the progress note, ACP documentation lacking the proper signatures is not legally acceptable. Despite the “.polst,” DotPhrase’s ability to accept electronic signatures, the majority of signatures were left blank because the residents were not given enough training.^22^

### Potential impact on claims and billing

A 2021 study on the topic showed that notes with more DotPhrase usage are invoiced at higher billing levels, even after accounting for patient complexity at presentation, note authorship, and note length.^1^

Thus, though EMR systems are useful for recording patient data, problems with usability cause inefficiencies that aggravate doctors and patients, as well as pose dangers to patient safety. These difficulties include process sequences that are tedious and repetitive and screen displays with unclear layouts and unnecessary information.^23^

## CONCLUSION

The incorporation of DotPhrases into the EMR has revolutionized clinical documentation by providing specialized and quick templates for health care providers. The advantages demonstrate increased EMR satisfaction, quicker note completion, and a higher level of documentation consistency. Templates with standardized formats ensure consistency that influences the quality of patient records, and DotPhrases simultaneously allow for personalization. Despite these benefits, problems can arise if DotPhrases are overused. Issues of overdocumentation, lack of uniformity, and physician exhaustion underline the importance of finding a balanced approach. Further, little is known about the drawbacks of DotPhrases concerning clinical documentation quality, time-saving, and physician burnout. Hospitals must develop an adequate management structure to optimize DotPhrases and CDS. These management responsibilities may include developing and then overseeing all DotPhrases in the EMR system. Further research on DotPhrases will allow us to lessen existing gaps in ACP and mitigate discrepancies in claims and billings.
